# Comparing genomic expression patterns across plant species reveals highly diverged transcriptional dynamics in response to salt stress

**DOI:** 10.1186/1471-2164-10-398

**Published:** 2009-08-25

**Authors:** Harkamal Walia, Clyde Wilson, Abdelbagi M Ismail, Timothy J Close, Xinping Cui

**Affiliations:** 1 Department of Plant Pathology, University of California, Davis, CA, USA; 2 Department of Botany and Plant Sciences, University of California, Riverside, CA, USA; 3 USDA-ARS, U. S. Salinity Laboratory, Riverside, CA, USA; 4 Crop and Environmental Sciences Division, International Rice Research Institute, Philippines; 5 Department of Statistics, University of California, Riverside, CA, USA

## Abstract

**Background:**

Rice and barley are both members of *Poaceae *(grass family) but have a marked difference in salt tolerance. The molecular mechanism underlying this difference was previously unexplored. This study employs a comparative genomics approach to identify analogous and contrasting gene expression patterns between rice and barley.

**Results:**

A hierarchical clustering approach identified several interesting expression trajectories among rice and barley genotypes. There were no major conserved expression patterns between the two species in response to salt stress. A wheat salt-stress dataset was queried for comparison with rice and barley. Roughly one-third of the salt-stress responses of barley were conserved with wheat while overlap between wheat and rice was minimal. These results demonstrate that, at transcriptome level, rice is strikingly different compared to the more closely related barley and wheat. This apparent lack of analogous transcriptional programs in response to salt stress is further highlighted through close examination of genes associated with root growth and development.

**Conclusion:**

The analysis provides support for the hypothesis that conservation of transcriptional signatures in response to environmental cues depends on the genetic similarity among the genotypes within a species, and on the phylogenetic distance between the species.

## Background

Phenotypic divergence can often be traced to gene expression variation. Gene expression variation resulting in phenotypic differences has been observed among individuals within a population, a species and across multiple species [1,2]. Although instances of phenotypic divergence and underlying expression variation were reported in the past, it was the advent of reliable microarray technology that enabled biologists to study expression variation at a whole-genome level and link it to phenotypic variation.

Several studies have focused on expression level variation among accession within the species and between species in *Drosophila*, primates, yeast and fish [1,3-5]. In the plant kingdom, however, comparative genomics expression analyses across species have been scarce. One noteworthy comparative interspecies experiment sampled different tissues and revealed a low correlation for conserved expression pattern between rice and *Arabidopsis *orthologs [6]. The comparison between these highly diverged model species representing the monocots and dicots was elaborated upon by measuring their responses to light and dark regimes in two tissue types [7]. The comparisons indicated expression level conservation to some degree during the light regime but not in changes triggered in response to darkness. The study further indicated a lack of conservation in response to light between shoot and root tissue, thus highlighting the significance of tissue level variation that exist in inter- and intra-species comparisons [8]. A comparison between two ecotypes of *A*. *thaliana *at early seedling stage identified about 8% of the transcriptome to be differentially expressed. Notably, genes with a difference in transcript abundance were enriched in genes responsive to environmental factors [9].

Studies comparing the transcriptomes of multiple species have provided insights on phenotypic variation and accompanying expression variation. However, the biological interpretation can be challenging due to large and diverse sets of genes which are expressed differently across species. A more targeted approach for multi-species comparative expression analysis is to determine how different species respond to a particular perturbation such as starvation, exposure to chemicals or a naturally occurring environmental stress. For instance in plants, the transcriptome of *Arabidopsis thaliana *and a salt-tolerant *Thellungiella halophila *have been compared for their response to salt stress [10,11]. An important finding of these studies was that the highly tolerant *Thellungiella halophila *had several salt-tolerance related genes constitutively expressed at a higher level compared to *A. thaliana *even in absence of stress. In the present study, the focus was on the differential response patterns of two distantly related plant species to salt stress. Two economically important members from the *Poaceae*, rice and barley, were selected and profiled with respect to the deviation in their transcriptional signatures upon exposure to salt stress.

Rice is highly susceptible to even moderate levels of salinity. In contrast, barley is among the most salt-tolerant cereal crops. Physiological approaches have been employed previously to characterize the phenotypic variation in salt tolerance for these two grass species [12]. The transcriptional responses of rice and barley genotypes were also characterized independently by several laboratories using various microarray platforms [13-15]. However, a detailed comparative genomics approach to elucidate how and why these species differ in their tolerance to salt stress at molecular level remains to be pursued.

Although a comparison between rice and barley at the expression level can be performed *in silico *using existing datasets, such an approach has some limitations. For instance, most of the experiments involve varying levels and durations of stress imposed on plants. Further, the developmental stage at which stress is imposed varies widely. These issues are expected to inflate the observed expression level variations. Since most of the previous studies only employ a single genotype of rice or barley for expression analysis, the generality of the interpretations is limited. In this study we have used an equivalent stress level, a cognate developmental stage, similar tissue, identical microarray technology and an identical statistical analysis to compare the expression differences between rice and barley as well as within the genera.

The basic research questions which we sought to address in the present study are as follows: How conserved or different are the responses of rice and barley genes upon exposure to salt stress? Is there any species specific activation or repression of pathway(s) which can be associated with salt-tolerance of barley or sensitivity of rice?

## Results and discussion

### Experimental design for comparative study

**Figure 1:**
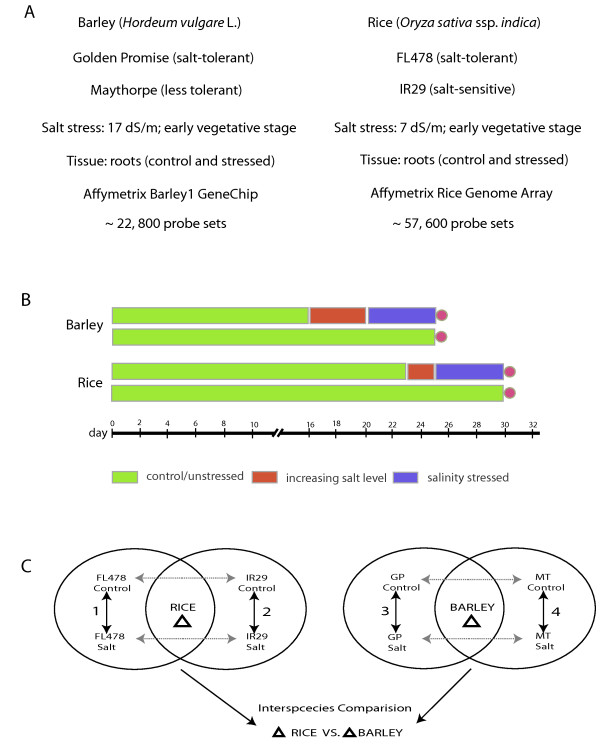
**Experimental design for salt stress treatment**. (A) Genotypic pairs with differences in salt tolerance and similar genetic backgrounds were selected for the transcriptome analysis using the Affymetrix GeneChip format. Comparable developmental stage and tissue were used for assay. (B) Stress treatment for rice and barley were implemented based on previous yield loss data. Green bar represents growth under unstressed/nutrient solution. Red bar indicates a gradual increase in salt levels. Blue bar represents growth under stress after reaching the final salt levels. Both control and stressed plants were harvested at the same time (circle). (C) Differential expression analysis comparisons for rice and barley. Current work focuses on comparisons 1 and 2 for rice and comparisons 5 and 6 for barley. Genes commonly regulated in both genotypes were considered the species specific responses. Genotypic comparisons 3, 4, 7 and 8 were not considered for present work.

The primary focus in designing the experiments was to assay biological materials at a comparable developmental phase and stress level for both species. We used two closely related rice genotypes IR29 (salt-sensitive) and FL478 (salt-tolerant) (Figure 1). The genotype FL478 is an F2-derived F8 recombinant inbred line whose phenology is similar to that of IR29 [16,17]. The tolerant rice genotype accumulated lower Na^+ ^in the roots and shoot tissue compared to sensitive IR29 when exposed to salt stress [see Additional file 1]. Further, the relative decrease in shoot and root biomass of salt stressed FL478 plants is significantly less compared to sensitive IR29. The genotypic pair used for barley was the salt-tolerant, irradiation-derived Golden Promise (GP) and its progenitor, Maythorpe (MT) [18]. The tolerant Golden Promise accumulates lower Na^+ ^in the shoot and root tissue compared to Maythorpe when grown under high salt conditions [19,20]. By employing rice and barley genotypic pairs with similar phenology we have attempted to minimize transcriptome differences resulting from differences in developmental phases. The current study used root material from plants in early tillering stage. Rice yield in particular is very sensitive to salinity during early tillering. The present study used matching tissue type (root tips) for rice and barley. Root samples were collected from plants growing under control and salt-stressed conditions. The salt stress levels selected for the both rice and barley were based on a yield loss of 60% for both, the sensitive rice (IR29) and sensitive barley (Maythorpe) [21,18]. We used the Rice Genome Array and Barley1 GeneChip from Affymetrix for transcriptional assay of rice and barley respectively.

### Orthologous gene representation between Rice Genome Array and Barley1 GeneChip

To compare the salinity stress response between rice and barley, we first determined the extent of rice genome representation on the Barley1 GeneChip. For this the Barley1 probe set annotations were exported from HarvEST:Barley [22]. The unique rice loci corresponding to the barley probe sets were determined based on BLASTX searches against the rice genome. Representation for 10,808 unique rice gene orthologs was found on the barley array. A parallel analysis for the Rice Genome Array using HarvEST:Rice yielded 52,660 rice loci. For this analysis, the splice variant derived gene models were ignored. Based on the described cross-platform mapping, Barley1 GeneChip represents orthologs for roughly one-fifth of the rice genes.

### Clustering analysis of rice and barley

We were interested in identifying genes that have similar and contrasting expression trajectories in response to salt-stress in rice and barley. The first method we used to address this question was the hierarchical clustering of orthologous pairs. The clustering analysis of the rice and barley dataset is expected to reveal genes whose response to salt stress is conserved across rice and barley. In addition, this analysis is likely to identify genes with distinct transcript trajectories, which may explain the differences in salinity tolerance between rice and barley.

**Figure 2:**
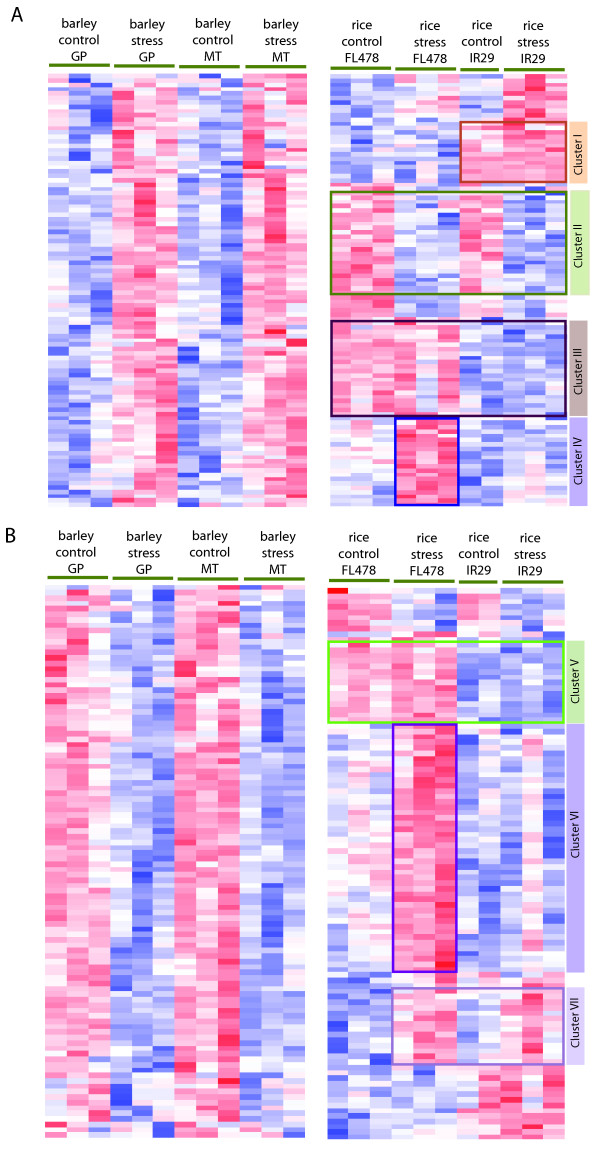
**Hierarchical clustering analysis of barley (left panels) and rice (right panel) genes that were responsive to salt stress in both barley genotypes (GP and MT)**. (A) Barley genes which were up-regulated in stressed root samples. The orthologous rice genes resolved into 4 clusters (I to IV). Cluster II gene members in rice have expression response trajectories opposite to their barley orthologs. (B) Genes which were repressed in barley genotypes during stress. The rice genes in Cluster VII have a response contrasting to their orthologs in barley. The cluster analysis was performed independently for barley and rice dataset. The heat map was generated based on relative expression of a given sample compared to the mean of the expression values across the samples for the given species (barley or rice). The red color is indicative of higher relative expression and the blue color represents lower expression than the mean value across the samples (white). The expression values were scaled from -3 to +3. P-value threshold for clustering genes was 0.001.

For the clustering analysis, 10,808 barley probe sets were included. These probe sets have a corresponding rice gene model and a representative probe set on rice array. Initially, hierarchical clustering was performed on the barley data set using these 10,808 probe sets. From these clusters were selected which have higher transcript abundance in both barley genotypes (GP and MT) when growing under salt-stressed conditions (Figure 2A, left panel). Clusters were then identified which were predominantly populated by genes with decreased transcript abundance in barley under stress (Figure 2B, left panel). To study the response of the rice orthologs of these two gene sets (induced and repressed sets in barley during stress), we performed the cluster analysis on the rice dataset for FL478 and IR29. The majority of the rice genes corresponding to the two barley genes sets were found to have distinct expression patterns. These genes were selectively extracted from the rice heat map and matched with their orthologous barley genes (Figure 2A and 2B). Based on these analyses several distinct clusters emerged which were annotated as Cluster I to VII [see Additional files 2, 3, 4, 5, 6, 7 and 8].

We have focused mainly on Cluster II and Cluster VII because they include genes which have contrasting responses between the rice and barley orthologs. Cluster II includes genes which have increased transcript levels in barley but were repressed in rice when exposed to salinity. Cluster VII is composed of genes whose transcript levels decrease in barley but increase in rice in response to salt stress. To obtain a broad functional categorization of these genes, the Functional Catalogue tool available from MIPS was used. The Cluster II gene set is enriched in cell rescue, defense and virulence (p-value < 0.006) and interaction with the environment (p-value < 0.002). A noteworthy member of this cluster is *LOW EXPRESSION OF OSMOTICALLY RESPONSIVE GENE 2 *(*LOS2*). Based on the cluster analysis, *LOS2 *is up-regulated in barley genotypes but down-regulated in rice upon exposure to high salt. *LOS2 *encodes a bifunctional enolase which is required for low-temperature response and acquired tolerance to freezing in *A. thaliana *[23]. Another member of Cluster II is *PHOSPHOLIPASE D DELTA*. Its regulation followed a similar pattern to that displayed by *LOS2*, i.e. it is also up-regulated in barley genotypes but down-regulated in rice upon exposure to high salt. It is worth noting that the *Arabidopsis *ortholog of *PLD-delta *is known to be involved in the H_2_O_2 _mediated programmed cell death (PCD). Activation of *PLD-delta *decreases H_2_O_2 _cell death and improves stress tolerance [24]. Further, to examine if any other members of the Cluster II are also responsive to PCD, *A. thaliana *orthologs of the rice genes were queried as to their response to PCD in the Response Viewer available from TAIR . At least 5 genes were found to be down-regulated and 2 genes up-regulated by PCD. *LOS2 *was also down-regulated during PCD in *A. thaliana*. Presuming functional conservation of *PLD-delta *among rice, barley and *Arabidopsis*, it is possible that decreased expression of *PLD-delta *makes rice roots more susceptible to cell death. The decreased expression of *LOS2 *and *PLD-delta *under salt stress is likely to make rice more sensitive to stress. In contrast, both these genes are induced in barley, potentially enabling the plant to ameliorate H_2_O_2_-mediated PCD-like conditions and improving stress tolerance.

Interestingly, there were no prominent gene clusters in rice and barley that had conserved expression patterns; however the cluster analysis described here primarily serves the purpose of a broad overview and lacks statistical robustness. Therefore, it is necessary to further examine the absence of conserved expression patterns between rice and barley by adopting a more statistically stringent differential expression analysis.

### Differential expression of rice and barley genes in response to salinity stress

Rice and barley data were analyzed separately to identify genes that are differentially expressed in response to salinity stress. The present work focuses only on comparing the response of rice and barley to stress. The response of rice or barley was defined to include genes which are commonly up-or down-regulated in both the genotypes of rice or barley (Figure 1C; comparisons 1 and 2 for rice and 4 and 5 for barley). Although genotypic comparisons within a species are important to understand expression level variation, we decided to focus on only the salt-stress responsive genes for the present work.

For calling differentially expressed genes in response to salinity, a threshold of 10% FDR in Statistical Analysis of Microarray (SAM) was used. The data preprocessing and statistical analysis is detailed in the Materials and Methods section. The number of probe sets identified by this statistical analysis for each genotype is listed in Table 1. The complete lists of differentially expressed genes for rice and barley are provided [see Additional files 9, 10, 11, 12, 13, 14, 15 and 16]. Fifty-eight probe sets were found to be commonly induced between the rice genotypes and 60 probe sets were repressed in both rice genotypes. In barley, the overlap between up-regulated and down-regulated probe sets was 250 and 179 respectively. Therefore, the conservation of expression responses was higher between barley genotypes than between rice. The barley genotypes, GP and MT are estimated to be more closely related than the rice genotypes IR29 and FL478. The number of differentially expressed genes in barley is likely to be an under-estimate because the barley array does not represent the complete genome.

**Table 1:** Number of probe sets differentially expressed in rice and barley

	**IR29**	**Os_common_**	**FL478**	**GP**	**Hv_common_**	**MT**
**up-reg**.	652	58	239	624	250	436

**down-reg**.	443	60	337	444	179	337

Differentially expressed genes were identified using 10% false discovery rate (FDR) cut-off

### Biological features extracted from GO analysis

**Figure 3:**
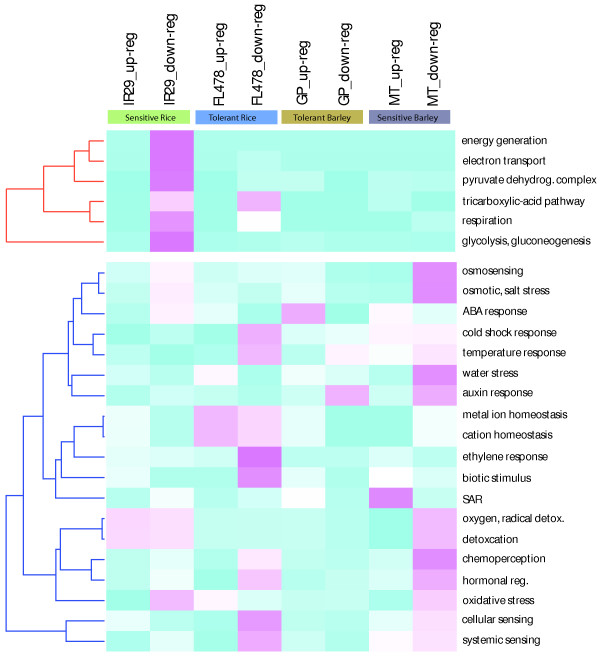
**Gene ontology (GO) analysis of genes differentially expressed in rice and barley**. The cluster colored with red branch represents the energy related GO sub-category. The blue branch represents two sub-categories cell rescue, defense and virulence, and interaction with environment. The clusters were obtained using the FunCat (MIPS) p-values for enrichment in a given gene set. The heat map was generated using -log (p-value) in R (heatmap). Blue color represents higher p-value hence less significant enrichment and magenta color represents higher enrichment (lower p-value). All p-values for enrichment were derived based on enrichment relative to whole genome gene sets.

We obtained the functional categorization of the differentially expressed genes for rice and barley in response to salinity stress. The GO analysis was performed separately for up-regulated and down-regulated genes for each genotype of rice and barley using the best BLASTX hit based *Arabidopsis *orthologs [see Additional files 17, 18, 19, 20, 21, 22, 23 and 24]. Three major GO sub-categories which predominantly highlighted interesting differences between rice and barley and/or between salt-tolerant and salt-sensitive genotypes are displayed in Figure 3. The figure shows two separate cluster branches color-coded red and blue. The red cluster represents genes involved in energy-related biological processes such as glycolysis, TCA pathway, and energy-generation among others. The most striking feature of this cluster is that the gene set down-regulated in IR29 in response to salt stress has a large number of genes associated with energy-related processes. Further, in FL478 the subcategories of TCA pathway and respiration are also over-represented in the down-regulated gene set. However, the barley genotypes GP and MT do not exhibit any notable decrease in energy generation and related processes in response to stress. This analysis indicates that energy generation related processes are more repressed in salt-sensitive rice compared to tolerant rice. Further, these processes did not exhibit a transcriptional repression in barley roots indicating that energy generation pathways are more sensitive to salt stress in rice than barley.

The blue cluster in Figure 3 includes selected sub-categories related to environmental stress response. In this cluster, a noteworthy distinction among tolerant and sensitive genotypes is that, in both rice and barley sensitive genotypes, a more significant proportion of the genes related to osmosensing and salt stress are down-regulated. The differentially regulated genes in the tolerant rice FL478 are highly enriched for genes involved in metal and cation homeostasis. A higher number of genes associated with oxygen radical detoxification are repressed in sensitive genotypes IR29 and MT. Plants activate detoxification genes to counter highly reactive and damaging species generated during oxidative stress. Reduced expression of detoxification associated genes in sensitive genotypes may distinguish the tolerant genotypes from their sensitive counterparts across species.

In addition to the categories represented on the heat map, some additional conspicuous subcategories were found. Genes mapping to the GO category pertaining to cell wall biogenesis showed a inverse response between rice and barley. Several genes in this category were repressed in barley. The situation was different for rice genotypes. The rice cell wall biogenesis category was very highly enriched in up-regulated genes but not many of the cell wall genes were down-regulated. The contrasting response between the rice and barley in the context of cell wall biogenesis is surprising considering that high salt levels in and around the roots are expected to retard growth and associated cell wall biogenesis activities in the root tissue.

### Comparing the transcriptional responses of rice and barley

Using the genes identified by differential expression analysis, we next sought to determine the degree to which the response of rice-barley orthologous pairs is shared on exposure to salt stress. For this analysis gene sets which are commonly regulated by both genotypes of rice and barley were used (Table 1). The response to salt stress as identified by differential expression analysis is highly divergent between rice and barley. Only a single gene Os05g13940 (a plasma-membrane linker protein) was found to be induced in both rice and barley. Four genes were commonly repressed in response to salt stress in both species.

These comparisons indicate that commonality between the two species at the transcription level is sparse at the threshold levels used in the current analysis for identifying salt responsive genes. The results however are consistent if viewed in context of the identified conserved responses between the genotypic pairs of both rice and barley. For instance only 24% of the genes induced in FL478 are shared with those induced in IR29 while only 17% of the genes repressed in FL478 are also regulated similarly by IR29. These numbers are unexpectedly low considering that IR29 and FL478 share at least half their genomic content because FL478 is an F2-derived RIL. The genotypic pair in barley is relatively more similar since GP and MT are known to differ at few haploblocks [20]. This higher genetic similarity may potentially explain the higher conservation observed between GP and MT. More than 50% of the genes up-regulated or down-regulated in GP are also similarly regulated in MT. Our analyses indicate an unexpectedly divergent response of barley and rice to salt stress. The observed divergence, as measured by differentially responsive orthologs, is consistent with the degree of genetic dissimilarity ascertained qualitatively.

### Salt-stress regulated transcriptome of wheat

To increase the power and generality of our comparative analysis approach, public databases were searched to find gene expression datasets of salt stress responses in other plant species. We selected a dataset generated for wheat roots response during salt stress using the Affymetrix Wheat Array [25]. These particular wheat data were generated using the same platform technology as with the rice and barley data. Therefore, the preprocessing and analysis of the wheat transcriptome data were performed using the same statistical protocols. This minimized introducing additional bias due to differences in the preprocessing (normalization and background correction) and differential expression analysis. The root tissue for the wheat data was collected from developmentally comparable plants but was exposed to higher salinity levels in case of stressed plants. Another important difference is that the wheat data were collected from the whole root tissue while the rice and barley data were generated from root tips. While a difference in stress levels and the tissue sampling are likely to result in gene expression changes, the comparisons can yield useful information.

The statistical analysis performed identified 1174 probe sets that were induced in response to salt stress. Based on best BLASTX hit criteria that was used previously for barley, these probe sets matched 583 unique rice loci. A total of 1304 probe sets were repressed under stress conditions in wheat. The down-regulated wheat probe sets corresponded to 784 rice loci. The unique rice loci filtered from the two wheat gene sets were used to make the interspecific comparisons. Twenty percent of the genes that are induced in barley (both GP and MT) were found to be induced in wheat. Similarly, a significant portion (34%) of the genes down-regulated in barley, were also down-regulated in wheat roots in response to salt stress. In striking contrast, less than 5% of the differentially expressed genes were shared between wheat and rice gene sets responding to salt stress. It is pertinent to note that conserved responses indicated by our comparison are likely underestimated as only the best BLASTX match rice loci were used for the analysis.

Regardless, the analysis clearly indicates that expression level response to high salt levels is conserved to a significant degree between wheat and barley but is highly diverged in rice. Interestingly, the expression divergence among rice, barley and wheat qualitatively correlates with evolutionary distance among these grass species. Rice and wheat diverged from a common ancestor ~46 million years ago (mya) [26] and one estimate of wheat and barley divergence indicates 11–15 mya [27]. Therefore sequence-based estimates separate rice and barley lineage by more than 40 million years. The observations herein are consistent with those reported for a comparative primate study. The primate study involved a comparison of expression profiles of multiple tissues among human, chimpanzee and macaque. The analysis revealed a higher expression-level similarity between human and chimpanzee than when either one was compared to evolutionarily distant macaque [4].

To ascertain if salt-sensitive rice has responses that are directly opposite to those of tolerant barley and wheat, we searched for genes which are up-regulated in rice but down-regulated in either barley or wheat. We identified six genes which were up-regulated in rice (both FL478 and IR29) and down-regulated in wheat. Similar comparison with barley identified four such genes. One of the two genes up-regulated in rice and down-regulated in both barley and wheat is *RESPONSE TO DESSICATION 22 *(*RD22*). Another gene is *ZRP4*, an O-methyltransferase originally identified in maize as a developmentally regulated gene strongly expressed in root endodermis [28]. The number of genes identified using the commonly regulated gene set for each species yielded very few genes with opposite response to salinity in roots. The scenario however, was surprisingly different when genes induced in IR29 were compared to genes repressed in wheat. Twenty nine genes were found to fulfill the criterion. No genes were found to be down-regulated in IR29 but activated in wheat. The 29 genes identified included several known key regulators of plants such as *NITRATE REDUCTASE 1 *(*NIA1*), *CBF1*, and two genes encoding for enzymes from flavonoid pathway *TRANSPARENT TESTA 7 *(*TT7*) and *CHALCONE SYNTHASE *(*CHS*).

### Salt-stress and root development

**Figure 4:**
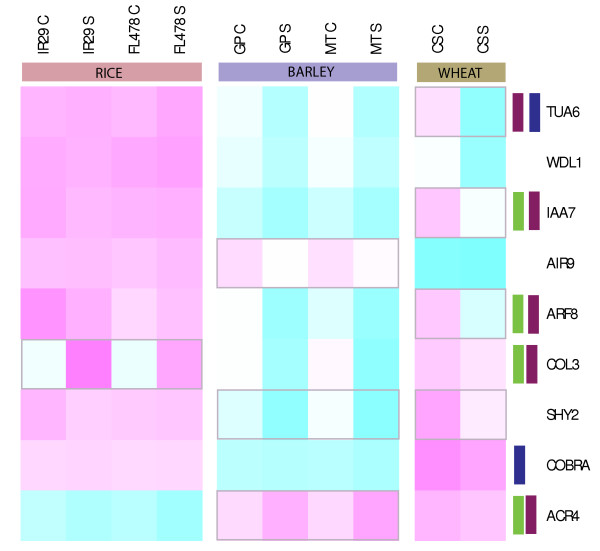
**Expression based clustering of root development genes in rice, barley and wheat**. The heatmap was generated using the mean expression values for the three *Triticeae *members. The pink color represents higher relative expression and the blue color indicates lower expression. Several of the genes are active only at growing points of primary roots and lateral root initiation sites in Arabidopsis thaliana. The highly localized expression domains were determined using cell-type specific microarray data. The green bars indicate columella root cap expression, while the magenta bar represents epidermis and lateral the root cap. The blue bar represents cortex specific expression.

During salt stress roots are not only exposed to higher cellular Na^+ ^but are also physically exposed to a high salt environment. In this context it was pertinent to determine how salt-stress regulates genes that are critical for normal root development programs. Identifying these stress-responsive developmental genes can provide insight into how different plant species with varying degree of stress tolerance change their developmental programs to adapt to stress. Several genes were identified which respond to salt stress and are known to determine the root architecture in plants. Some of these genes are represented on the heat map in Figure 4 which displays their relative expression across rice, barley and wheat.

Differential analysis identified *CONSTAN-LIKE 3 *(*COL3*) to be highly induced in rice but repressed in barley and wheat under stress. *COL3 *has been reported to promote lateral root formation [29]. We find it intriguing that *COL3 *and several other genes involved in root development which are expected to be down-regulated in response to salt stress are either unchanged or slightly induced in rice roots. Most of these genes are down-regulated in barley and wheat during salt stress. The information on the salt regulated root-developmental genes was further refined by employing the rich spatial transcriptomic resources that are available for model plant *Arabidopsis*. The expression profiles of the *Arabidopsis *orthologs of these genes were studied by analyzing the cell-type specific data for roots under control and short-term salt stress [30]. Several of the genes identified from rice, barley and wheat analysis have enhanced cell-type specific expression. These genes were primarily expressed in columella root cap (indicated by green bar) and epidermis and lateral root cap (magenta bar) in *Arabidopsis *when each of the cell type was teased apart and assay separately. Both these root tissue types are critical sites for root growth. *Arabidopsis *orthologs of at least three members of the cluster (*COBRA*, *ARF8 *and *TUA6*) are down-regulated in response to salt stress.

Collectively, these data indicate that several of the root development genes that populate the cluster are likely to have high cell-type specificity of expression for root growing points in cereals. These genes are known critical regulators of root growth. In the current analysis they were repressed during salt stress in barley, wheat and *Arabidopsis *but not in rice. This interpretation, although based on a small and selected set of genes, is consistent with the genome-wide observation that transcriptome dynamics in rice roots are highly diverged from those of barley and wheat. This particular response could possibly result if highly sensitive rice is programmed to promote root growth in an attempt to grow away from high salt zone in natural environments. Alternatively, rice roots could continue to grow at the lower salinity levels they were exposed to in the present experiment while the relatively higher stress levels in barley and wheat represses root growth promoting genes.

### Salt stress and auxin regulation in roots

The comparative multi-species transcriptional analysis in the present study identified several key root development regulators which respond differentially between the rice and relatively more tolerant barley and wheat in the presence of high salt. Since auxin is central to root growth and development, data sets supporting the study were further explored for evidence which may link perturbed auxin homeostasis to differential response of developmental genes during salt stress. We found several auxin-related genes to be responsive to salt stress in barley and wheat but only to a limited degree in rice. The GO analysis also highlighted the fact that gene sets down-regulated in barley in response to salt stress are more enriched in the auxin-related genes than the responsive gene sets in rice (Figure 3). The distinct contrast between rice and barley in the context of root developmental genes and auxin-related genes could be interpreted as either a lack of response or a delayed response of rice when exposed to high salt. This apparent lack of adjustment in root developmental programs upon exposure to salinity could potentially explain the salt-sensitivity of rice relative to barley and wheat.

It is useful and important to also consider the results in a physiological context such as differences in ion transport among the three species. More specifically, the rate of Na^+ ^transport and Na^+ ^concentration likely differs among these three species. In light of this and other ion-transport linked possibilities, the microarray data were reexamined specifically for responses of Na^+ ^transporter encoding genes in the rice genome. The expression of most of the Na^+ ^transporter genes was found to be highly variable across replicates as well as treatments (data not shown). Similar observations were also made from the barley dataset. The role of ion transporters in overall uptake of ions to the shoot may vary with species. Higher Na^+ ^concentrations in shoot tissue in rice is thought to result from partly from transpirational bypass flow in addition to net Na^+ ^uptake [31]. Thus, on a speculative note, the regulation of the expression of Na^+ ^transporter encoding genes in response to salinity stress may play a more limited role in tolerance, at least in rice. The regulation of overall root development and root architecture in rice may play a major role in tolerance, too. These analyses also highlight the importance of more detailed physiological measurements such as Na^+^, K^+^, Cl^- ^ion levels in the different tissue types and several time points for transcript analysis in future experiments.

## Conclusion

Comparative genomics is a useful approach for identifying biological features which are evolutionarily conserved across species and those which are characteristics of a particular species. Aided by publicly available expression data sets for wheat and *Arabidopsis*, this study compared the root transcriptome of two important cereal species in response to salt stress. While we did not identify any conservation in expression level response, the analysis successfully revealed several key biological features that distinguish rice from barley and wheat which are relatively more closely related. The study's findings may explain the phenotypic difference between rice and barley. We propose that under salt stress, rice is unable to repress key biological activities associated with growth such as root development genes, cell wall biogenesis genes and auxin-regulated genes. In contrast, genes associated with these three subcategories are suppressed in barley and also in wheat. It should be note that while these features clearly distinguish the response of rice from barley, it is difficult to ascertain if these features are the cause or the effect of inherent salt-sensitivity of rice. What is needed is a detailed study of root growth and development, and root architecture coupled with a high-resolution transcriptional analysis to help ascertain the mechanism of salt adaptation in cereals. The interpretations from the current study should be taken with some degree of caution, though, as they are limited to the analysis of the roots of these species at one developmental stage. Future work will focus on the shoot tissue and aim for a higher spatial and developmental resolution in several genotypes across multiple species. Such an approach will elucidate orthologous transcriptional networks required for response to environmental stress in a wide array of related species.

## Methods

Rice genotypes IR29 and FL478 were originally obtained from G.B. Gregorio at IRRI, Philippines and seed was multiplied by Linghe Zeng at U. S. Salinity Laboratory, Riverside. Seed for barley genotypes Golden Promise was a gift from Peggy Lemaux (University of California, Berkeley) and Maythorpe was obtained from the National Small Grains Collection, USDA-ARS, Aberdeen, Idaho. The plants were cultured for expression studies at the U.S. Salinity Laboratory, USDA-ARS, Riverside, CA.

### Rice salinity stress treatment

Rice plants were grown in large tanks (122 × 61 × 46 cm) filled with sand and irrigated with Yoshida solution. The seedlings were grown with nutrient solution for 22 d after germination. Salinity stress treatment was applied by adding NaCl and CaCl_2 _(5:1 molar concentration) in 2 steps to a final EC_w _of 7.4 dSm^-1 ^(~60 mM NaCl) over a period of 3 d to avoid a sudden osmotic stress. The EC_w _of control tanks was 1.1 dSm^-1^. The nutrient solution pH was maintained between 5.0 and 6.5 by adding sulfuric acid. Plants were exposed to salt stress for 5 more days. The rationale was to allow the plants to adapt to high salinity and capture the more relevant adaptive responses. Roots (2 cm of root tips) were harvested and snap frozen for RNA extraction 30 d after germination. The rice plants from the control tanks were also harvested at the same time. The plants were in early tillering stage of development at the time of harvest. Three biological replicates were obtained for FL478 and IR29 from both control and high salinity sand tanks. The air temperature ranged from 32°C to 45°C during the day and between 19°C to 22°C at night. Relative humidity ranged from 40% to 80%.

### Barley salinity stress treatment

Germinated barley seeds of Golden Promise and Maythorpe were transferred to Speedling transplant trays (polystyrene trays with cells) floated on aerated half-strength Hoagland's solution with double iron (50 gL^-1^) in two large (700 L) metal containers in the greenhouse. Sixteen days after germination, the plants in one container were exposed to a gradually increasing salt stress. The final EC_w _17 dSm^-1 ^(~150 mM NaCl) was achieved by gradually increasing the salt concentration during a period of 5 days. A Na:Ca molar ratio of 10:1 was maintained during each increment of salinity level by adding CaCl_2_. The system was allowed to stabilize for 5 more days and then roots (2 cm of root tips) of stressed and control plants for both genotypes were harvested and snap frozen. Roots from ~15 plants were harvested for each genotype to constitute a biological replicate. Material from 3 independent biological replicates was obtained for RNA extraction.

The root tissue harvest stage for both species was during the early tillering when rice yield is highly sensitive to salt stress. The salt stress levels selected for the both rice (7.4 dSm^-1^/~60 mM NaCl) and barley (17 dSm^-1^/~150 mM NaCl) were based on past experiments which indicated a yield loss by 60% for both, the sensitive rice (IR29) and sensitive barley (Maythorpe) [21,18].

### Wheat salinity stress treatment

Wheat microarray data included in this analysis was generated by Mott and Wang (2007). We used the wheat cv. Chinese Spring root sample microarray data. The salinity level reported in the wheat experiment was a gradual increment (6 dS/m) to reach a final EC of 30 dS/m. The RNA for the microarray was obtained from whole root tissue of 42 d old plants. The wheat data used in the current analysis was generated from a relatively higher salt stress treatment. Further the plants were sampled in a later developmental stage than rice and barley. The rice and barley dataset were generated using the RNA from root tips while the wheat data was obtained from whole roots. These differences are significant and are expected to influence the transcriptional responses to salinity stress among the three species. However, the comparison of wheat data with rice and barley indicates that such an analysis can provide useful information.

### RNA preparation and GeneChip hybridization

RNA extraction, clean-up, labeling and hybridization to GeneChips for rice and barley root samples was done as described in Walia et al., 2005. Briefly, total RNA was isolated from root tissue using the TRIzol reagent. The RNA was cleaned by passing through an RNAeasy spin column (Qiagen, Chatsworth, CA) and on-column DNase1 treated according to manufacturer's protocol. The RNA quality was assessed using the RNA Lab-On-A-Chip (Caliper technologies Corp., Mountain View, CA) on a Agilent Bioanalyzer 2100 (Agilent Technologies, Palo alto, CA). Further labeling and hybridization steps were performed as recommended by Affymetrix, Inc. (Affymetrix Genechip Expression Analysis Technical Manual, Affymetrix, INC., Santa Clara, CA). Each biological replicate was hybridized to an array to obtain a total of 3 replicates for each genotype and treatment. The only exception was IR29 control samples for which for which only 2 biological replicates are available.

### Data analysis

Scanned GeneChip images from rice and barley were examined for visual aberrations. Images with visual defects were discarded and labeled cRNA was hybridized to fresh GeneChips. Further preprocessing and analysis was performed using the .CEL files. The .CEL files were imported into RMA [32] for further processing. The background adjustment and quantile normalization were performed using the default settings. The log-transformed values from RMA were imported into Significance Analysis of Microarrays (SAM) software [33]. To identify salt stress responsive genes we performed differential expression analysis using the two-class unpaired selection in SAM. The test statistics used was the T-statistic. The arbitrary cut-off for calling differential expression was set at 10% FDR (q-value). We did not use a fold change threshold for analysis using SAM. The rice and barley dataset were analyzed as described above. Publicly available wheat (E-MEXP-971; ArrayExpress EBI) and *Arabidopsis *(GSE7642; NCBI GEO) datasets were also analyzed independently using the described preprocessing and differential expression analysis.

### Probe set Annotation

The probe sets for rice, barley and wheat were obtained using the HarvEST:Rice (ver. 1.10), HarvEST:Barley (ver. 1.72) and HarvEST:Wheat (ver. 1.52) annotation tool publicly available at [34]. The software provides the best BLASTX hit against UniProt, TIGR Rice, and TAIR *Arabidopsis *database with a cut-off threshold of E-20. The minimum number of probes matched selected for the annotation was 11. The annotation for *Arabidopsis *probe sets was obtained from the BAR tool [35].

### Hierarchical clustering analysis

Unsupervised hierarchical clustering analysis was performed separately for rice and barley datasets. The threshold for calling significant gene clusters was set to a p-value of 0.001. Pearson correlation was used for placing neighboring probe sets. The Gene Ontology (GO) heat cluster was generated using the -log (p-value) for each sub-category (Figure 3). The color code for the heat map displays pink for lower p-value (more significant enrichment) and blue represents low or no relative enrichment of a category for a given gene set.

## Availability and requirements

The rice and barley data are available through the NCBI portal GEO under series GSE13735 and GSE6325. The list of differentially expressed genes and GO is available as Additional files. Enhanced probeset annotations for rice, barley and wheat were generated using the publicly accessible annotation tools available at .

## Authors' contributions

HW contributed in the design of the experiment, cultured the plants, analyzed the array data, and drafted the manuscript. CW designed the experiment, cultured the plants, performed ion analysis, and provided significant input to the manuscript. AMI is the co-principal investigator on the project and had major input in the design of the experiment. XC and TJC are the principal investigators of the grants that funded the research and had input in the design of experiment, data interpretation and manuscript preparation.

## Supplementary Material

Additional file 1 **Genes down-regulated in FL478 roots** Click here for file

Additional file 2 **Genes up-regulated in FL478 roots** Click here for file

Additional file 3 **Genes down-regulated in IR29 roots** Click here for file

Additional file 4 **Genes up-regulated in IR29 roots** Click here for file

Additional file 5 **Genes down-regulated in Golden Promise roots** Click here for file

Additional file 6 **Genes up-regulated in Golden Promise roots** Click here for file

Additional file 7 **Genes down-regulated in Maythorpe roots** Click here for file

Additional file 8 **Genes down-regulated in Maythorpe roots** Click here for file

Additional file 9 **Figure **2:**Cluster 1** Click here for file

Additional file 10 **Figure **2:**Cluster 2** Click here for file

Additional file 11 **Figure **2**:Cluster 3** Click here for file

Additional file 12 **Figure **2**:Cluster 4** Click here for file

Additional file 13 **Figure **2**:Cluster 5** Click here for file

Additional file 14 **Figure **2**:Cluster 6** Click here for file

Additional file 15 **Figure **2**:Cluster 7** Click here for file

Additional file 16 **GO for FL478 down-regulated genes** Click here for file

Additional file 17 **GO for FL478 up-regulated genes** Click here for file

Additional file 18 **GO for Golden Promise down-regulated genes** Click here for file

Additional file 19 **GO for Golden Promise up-regulated genes** Click here for file

Additional file 20 **GO for IR29 down-regulated genes** Click here for file

Additional file 21 **GO for IR29 up-regulated genes** Click here for file

Additional file 22 **GO for Maythorpe down-regulated genes** Click here for file

Additional file 23 **GO for Maythorpe up-regulated genes** Click here for file

## References

[B1] Brem RB, Yvert G, Clinton R, Kruglyak L (2002). Genetic dissection of transcriptional regulation in budding yeast. Science.

[B2] Townsend JP, Cavalieri D, Hartl DL (2003). Population genetic variation in genome-wide gene expression. Mol Biol Evol.

[B3] Jin W, Riley RM, Wolfinger RD, White KP, Passador-Gurgel G, Gibson G (2001). The contributions of sex, genotype and age to transcriptional variance in Drosophila melanogaster. Nat Genet.

[B4] Caceres M, Lachuer J, Zapala MA, Redmond JC, Kudo L, Geschwind DH, Lockhart DJ, Preuss TM, Barlow C (2003). Elevated gene expression levels distinguish human from non-human primate brains. Proc Natl Acad Sci.

[B5] Oleksiak MF, Roach JL, Crawford DL (2005). Natural variation in cardiac metabolism and gene expression in Fundulus heteroclitus. Nat Genet.

[B6] Ma L, Chen C, Liu X, Jiao Y, Su N, Li L, Wang X, Cao M, Sun N, Zhang X, Bao J, Li J, Pedersen S, Bolund L, Zhao H, Yuan L, Wong GK, Wang J, Deng XW, Wang J (2005). A microarray analysis of the rice transcriptome and its comparison to Arabidopsis. Genome Res.

[B7] Jiao Y, Ma L, Strickland E, Deng XW (2005). Conservation and divergence of light-regulated genome expression patterns during seedling development in rice and arabidopsis. Plant Cell.

[B8] Whitehead A, Crawford D (2005). Variation in tissue-specific gene expression among natural populations. Genome Biol.

[B9] Zhang X, Byrnes JK, Gal TS, Li W-H, Borevitz JO (2008). Whole genome transcriptome polymorphisms in Arabidopsis thaliana. Genome Biol.

[B10] Zeng L, Shannon MC (2000). Salinity effects on the seedling growth and yield components of rice. Crop Sci.

[B11] Ueda A, Kathiresan A, Bennet J, Takabe T (2006). Comparative transcriptome analyses of barley and rice under salt stress. Theoretical and Applied Genetics.

[B12] Ozturk ZN, Talame V, Deyhoyos M, Michalowski CB, Galbraith DW, Gozukirmizi N, Tuberosa R, Bohnert HJ (2002). Monitoring large-scale changes in transcript abundance in drought- and salt-stressed barley. Plant Mol Biol.

[B13] Kawasaki S, Borchert C, Deyholos M, Wang H, Brazille S, Kawai K, Galbraith D, Bohnert HJ (2001). Gene expression profiles during the initial phase of salt stress in rice. Plant Cell.

[B14] Wong CE, Li Y, Labbe A, Guevara D, Nuin P (2006). Transcriptional profiling implicates novel interactions between abiotic stress and hormonal responses in *Thellungiella*, a close relative of *Arabidopsis*. Plant Physiol.

[B15] Gong QQ, Li PH, Ma SS, Rupassara SI, Bohnert HJ (2005). Salinity stress adaptation competence in the extremophile *Thellungiella halophila *in comparison with its relative *Arabidopsis thaliana*. Plant J.

[B16] Bonilla P, Dvorak J, Mackill D, Deal K, Gregorio G (2002). RFLP and SSLP mapping of salinity tolerance genes in chromosome 1 of rice (Oryza sativa L.) using recombinant inbred lines. Philippine J Agric Sci.

[B17] Walia H, Wilson C, Condamine P, Liu X, Ismail AM, Zeng L, Wanamaker SI, Mandal J, Xu J, Cui X, Close TJ (2005). Comparative transcriptional profiling of two contrasting rice genotypes under salinity stress during the vegetative growth stage. Plant Physiol.

[B18] Forster BP (2001). Mutation genetics of salt tolerance in barley: an assessment of Golden Promise and other semi-dwarf mutants. Euphytica.

[B19] Wei W, Bilsborrow PE, Hooley P, Fincham DA, Lombi E, Forster BP (2003). Salinity induced differences in growth, ion distribution and partitioning in barley between the cultivar Maythorpe and its derived mutant Golden Promise. Plant and Soil.

[B20] Walia H, Wilson C, Condamine P, Ismail AM, Xu J, Cui X, Close TJ (2007). Array-based genotyping and expression analysis of barley cv. Maythorpe and Golden Promise. BMC Genomics.

[B21] Zhang W, Wang C, Qin C, Wood T, Olafsdottir G, Welti R, Wang X (2003). The oleate-stimulated phospholipase D, PLDdelta, and phosphatidic acid decrease H_2_O_2_-induced cell death in Arabidopsis. Plant Cell.

[B22] Close TJ, Wanamaker S, Roose ML, Lyon M (2007). HarvEST: An EST Database and Viewing Software. Methods Mol Biol.

[B23] Lee H, Guo Y, Ohta M, Xiong L, Stevenson B, Zhu J-K (2002). LOS2, a genetic locus required for cold-responsive gene transcription encodes a bi-functional enolase. EMBO J.

[B24] Zhang W, Wang C, Qin C, Wood T, Olafsdottir G, Welti R, Wang X (2003). The oleate-stimulated phospholipase D, PLDδ, and phosphatidic acid decrease H_2_O_2_-induced cell death in Arabidopsis. Plant Cell.

[B25] Mott IW, Wang RCC (2007). Comparative transcriptome analysis of salt-tolerant wheat germplasm lines using wheat genome arrays. Plant Science.

[B26] Salse J, Bolot S, Throude M, Jouffe V, Piegu B, Quraishi UM, Calcagno T, Cooke R, Delseny M, Feuillet C (2008). Identification and characterization of shared duplications between rice and wheat provide new insight into grass genome evolution. Plant Cell.

[B27] Ramakrishna W, Dubcovsky J, Park YJ, Busso C, Emberton J, SanMiguel P, Bennetzen JL (2002). Different types and rates of genome evolution detected by comparative sequence analysis of orthologous segments from four cereal genomes. Genetics.

[B28] Held BM, Wang H, John I, Wurtele ES, Colbert JT (1993). An mRNA putatively coding for an *O*-methyltransferase accumulates preferentially in maize roots and is located predominantly in the region of the endodermis. Plant Physiol.

[B29] Datta S, Hettiarachchi GH, Deng XW, Holm M (2006). *Arabidopsis *CONSTANS-LIKE3 is a positive regulator of red light signaling and root growth. Plant Cell.

[B30] Dinneny JR, Long TA, Wang JY, Jung JW, Mace D, Pointer S, Barron C, Brady SM, Schiefelbein J, Benfey PN (2008). Cell Identity Mediates the Response of Arabidopsis Roots to Abiotic Stress. Science.

[B31] Yeo AR, Yeo ME, Flowers TJ (1987). The contribution of an apoplastic pathway to sodium uptake by rice roots in saline conditions. Journal of Experimental Botany.

[B32] Irizarry RA, Hobbs B, Collin F, Beazer-Barclay YD, Antonellis KJ, Scherf U, Speed TP (2003). Exploration, Normalization, and Summaries of High Density Oligonucleotide Array Probe Level Data. Biostatistics.

[B33] Tusher V, Tibshirani R, Chu G (2001). Significance analysis of microarrays applied to the ionizing radiation response. Proc Natl Acad Sci.

[B34] HarvEST Home Page http://harvest.ucr.edu.

[B35] The Bio-Array Resource for Arabidopsis Functional Genomics http://bar.utoronto.ca/.

